# Influence of Sex, Race and Ethnicity, and Deprivation on Survival and Completion of the Fontan Pathway for Children With Functionally Single Ventricle Heart Disease

**DOI:** 10.1161/CIRCULATIONAHA.124.069779

**Published:** 2024-07-16

**Authors:** Rachel L. Knowles, Deborah Ridout, Qi Huang, Rodney C. Franklin, Anna N. Seale, Hannah Bellsham-Revell, Ferran Espuny-Pujol, Katherine L. Brown

**Affiliations:** 1 Population, Policy and Practice Research and Teaching Department, Great Ormond Street Institute of Child Health (R.L.K., D.R.), University College London, UK.; 2 Clinical Operational Research Unit (Q.H., F.E.-P.), University College London, UK.; 3 Paediatric Cardiology, Royal Brompton and Harefield National Health Service Foundation Trust, London, UK (R.C.F.).; 4 Cardiology, Birmingham Women’s and Children’s National Health Service Foundation Trust, UK (A.N.S.).; 5 Paediatric Cardiology, Evelina London Children’s Hospital, UK (H.B.-R.).; 6 Cardiac, Critical Care and Respiratory Division, Great Ormond Street Hospital for Children National Health Service Foundation Trust, London, UK (K.L.B.).

**Keywords:** child, Fontan procedure, heart diseases, social determinants of health

The influence of key social determinants of health (sex, race and ethnicity, and local area deprivation) on childhood mortality and timing of completion of palliative stage 3 Fontan-type surgery were investigated for a nationally representative cohort of children with functionally single ventricle hearts (f-SV). The UK National Health Service provides universal free health care; therefore, ability to pay should not directly influence service access. Nevertheless, sociocultural factors and lifestyle factors may lead to outcome disparities.

The study included children born between 2000 and 2018 who underwent palliative procedures for f-SV recorded in the mandatory National Congenital Heart Disease Audit.^1^ Primary and secondary outcomes were childhood survival and completion of Fontan-type surgery. Five-year survival was 72.1%. Institutional review board approval was obtained from the National Health Service Research Ethics Committee (approval 18/LO/1688) alongside Confidentiality Advisory Group support to waive consent (waiver 17/CAG/0071). Data supporting this study are available from the National Institute for Cardiac Outcomes Research (www.nicor.org.uk/researchers).

Participants were classified on the basis of biologic sex at birth. Race and ethnicity was recorded using National Health Service coding, which emphasizes self-reporting, and grouped using nationally recognized categories (White, Black, Asian, mixed ethnicity, or other). Residential postal code was converted into Index of Multiple Deprivation scores for English children and grouped into quintiles from 1 (most deprived) to 5 (least deprived). Children were classified into 8 f-SV subgroups (Table).^1^

**Table.**
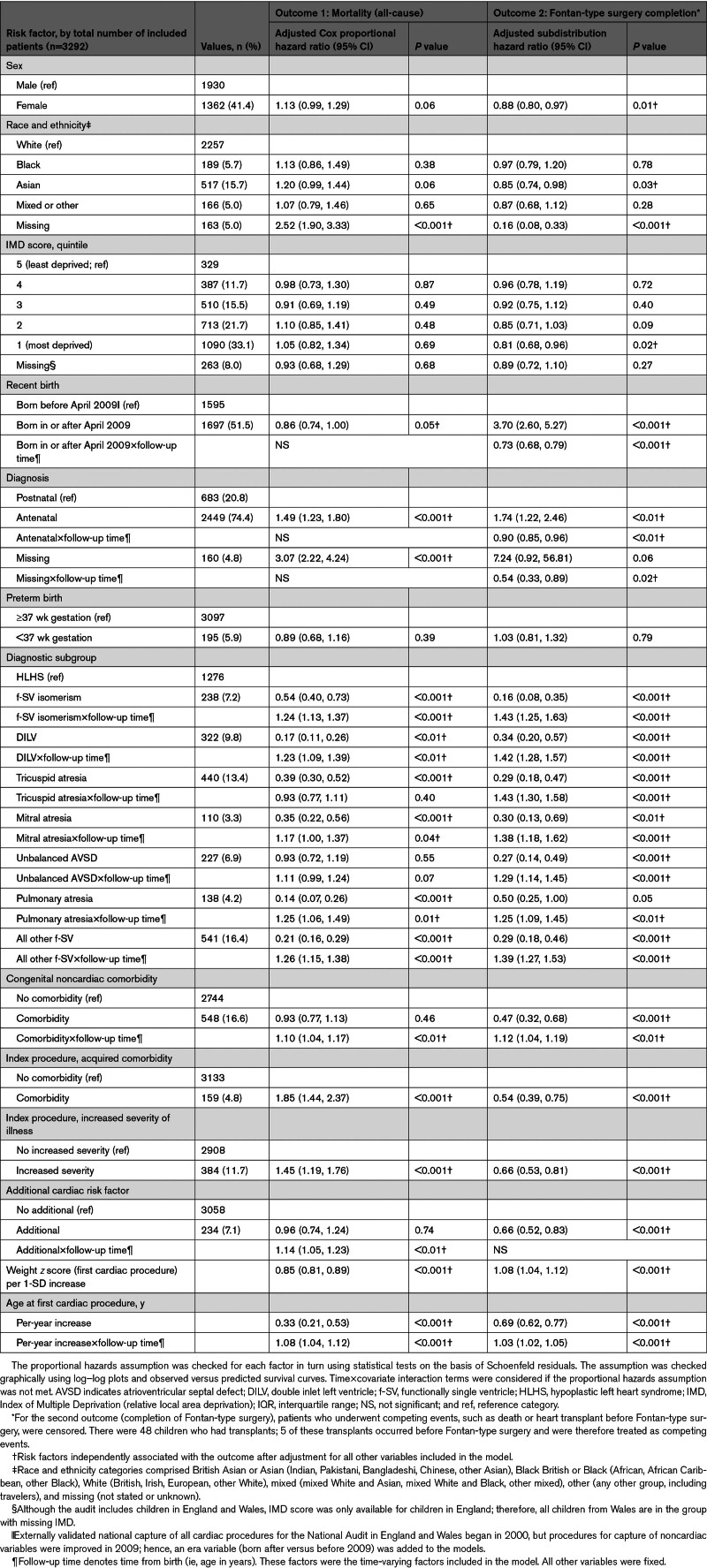
Primary (All-Cause Mortality) and Secondary (Fontan-Type Surgery Completion) Outcomes in Children With Functionally Single-Ventricle Disease by Risk Factor

Univariable and multivariable Cox proportional hazards models were developed to explore relationships between childhood survival and sex, race and ethnicity, and area deprivation, adjusting for prespecified clinical factors. Children who survived transplantation were included as survivors. Comparisons between risk factors and likelihood of achieving Fontan-type surgery used 1-way ANOVA with Bonferroni correction. The association between risk factors (Table) and completing Fontan-type surgery was investigated using multivariable Fine-Gray regression; competing events were death and cardiac transplantation. The National Congenital Heart Disease Audit is a procedure-based data set; therefore, it excludes patients who did not undergo procedures, and cannot capture differences in survival before intervention or because of pregnancy termination. Moreover, it lacks information to investigate geographic factors, including distance to specialist care.

Of 3292 children (Table), 1362 (41.4%) were girls, 195 (5.9%) were preterm, and f-SV was diagnosed antenatally in 2449 (74.4%). Girls were more likely to have f-SV isomerism and additional cardiac risk factors than boys. Most children were White (68.6%) or Asian (15.7%). Asian children were more likely to have f-SV isomerism and congenital noncardiac comorbidities, acquired comorbidity, or more severe illness at first procedure. Children from more deprived areas were more likely to have noncardiac congenital comorbidities.

No evidence of differences in survival to 18 years by sex, race and ethnicity, or area deprivation was found after adjusting for clinical factors (Table). In multivariable competing risk models, adjusted for prespecified clinical and time-varying factors (Table), female sex was associated with lower likelihood of completing Fontan-type surgery (adjusted subhazard ratio, 0.88 [95% CI, 0.80, 0.97]). Children from the most deprived quintile were significantly less likely to complete Fontan-type surgery than those in the least deprived quintile (adjusted subhazard ratio, 0.81 [95% CI, 0.68, 0.96]). Asian children were less likely to complete Fontan-type surgery than White children (adjusted subhazard ratio, 0.85 [0.74, 0.98]); this was partly explained by higher preoperative mortality rates (adjusted subhazard ratio, 1.24 [95% CI, 1.02, 1.52]).

For 1582 children (48.1%) who completed Fontan-type surgery, median age at completion was 4.52 (interquartile range, 3.68, 5.60) years, and median weight was 16.0 kg (interquartile range, 14.3, 18.4). Completion rates varied by f-SV subgroup. At all ages, girls were less likely to complete Fontan-type surgery than boys, and Asian or Black children were less likely to complete Fontan-type surgery than White children. Girls and Asian children underwent Fontan-type surgery at higher median ages compared with boys and White children, and children in the most deprived quintile were operated at an older median age than those in the least deprived quintile (*P*<0.05; 1-way ANOVA and post hoc Bonferroni tests).

Despite marked improvements in childhood survival with f-SV, disparities related to social determinants have been reported.^2,3^ Disadvantaged populations can experience multiple barriers to accessing high-quality care, including inequitable provision, structural racism, and geography. Within the UK context of universal free access to health care, no association was found between childhood survival and sex, race and ethnicity, or deprivation. A recent US analysis suggested that enhanced health insurance coverage improved access to care and reduced racial and ethnic disparities in mortality rates.^4^

The optimal age for stage 3 Fontan-type surgery depends on multiple clinical factors. In this study, female sex, Asian race, and area deprivation were associated with lower likelihood of completing Fontan-type surgery after adjustment for f-SV subtype and comorbidities. In these subgroups, Fontan-type surgery was performed at a higher median age and lower weight *z* score. Evidence that growth in patients with f-SV is modifiable suggests that additional effort could be focused on optimizing interstage growth.^5^ Whether the observed disparities are also associated with adult mortality rates, exercise performance, or neurodevelopment merits further investigation.

## ARTICLE INFORMATION

### Acknowledgments

The authors thank the data managers and audit leads at the UK centers, the data management team at the National Institute for Cardiovascular Outcomes Research for providing the National Congenital Heart Disease Audit data for this study, and Natasha Khan, Victor Tsang, and Caner Salih for their comments on the manuscript.

### Sources of Funding

This study was funded by the British Heart Foundation (grant PG/17/88/33401). Prof Brown received support from the National Institutes of Health and Care Research Biomedical Research Centre at Great Ormond Street Hospital.

### Disclosures

Prof Brown and Dr Franklin sit on the steering committee of the National Congenital Heart Disease Audit.
